# Malariometric Survey of Ibeshe Community in Ikorodu, Lagos State: Dry Season

**DOI:** 10.1155/2013/487250

**Published:** 2013-05-23

**Authors:** Oluwagbemiga O. Aina, Chimere O. Agomo, Yetunde A. Olukosi, Hilary I. Okoh, Bamidele A. Iwalokun, Kathleen N. Egbuna, Akwaowo B. Orok, Olusola Ajibaye, Veronica N. V. Enya, Samuel K. Akindele, Margaret O. Akinyele, Philip U. Agomo

**Affiliations:** Malaria Research Laboratory, Nigerian Institute of Medical Research, 6 Edmond Crescent, P.M.B 2013, Yaba, Lagos 101212, Nigeria

## Abstract

Malariometric surveys generate data on malaria epidemiology and dynamics of transmission necessary for planning and monitoring of control activities. This study determined the prevalence of malaria and the knowledge, attitude, and practice (KAP) towards malaria infection in Ibeshe, a coastal community. The study took place during the dry season in 10 villages of Ibeshe. All the participants were screened for malaria. A semistructured questionnaire was used to capture sociodemographic data and KAP towards malaria. A total of 1489 participants with a mean age of 26.7 ± 20.0 years took part in the study. Malaria prevalence was 14.7% (95% CI 13.0–16.6%) with geometric mean density of 285 parasites/*μ*L. Over 97% of participants were asymptomatic. Only 40 (2.7%) of the participants were febrile, while 227 (18.1%) were anemic. Almost all the participants (95.8%) identified mosquito bite as a cause of malaria, although multiple agents were associated with the cause of malaria. The commonest symptoms associated with malaria were hot body (89.9%) and headache (84.9%). Window nets (77.0%) were preferred to LLIN (29.6%). Malaria is mesoendemic in Ibeshe during the dry season. The participants had good knowledge of symptoms of malaria; however, there were a lot of misconceptions on the cause of malaria.

## 1. Introduction

Malaria remains one of the greatest causes of morbidity and mortality in the world. Global estimate shows that there are about 219 million cases of malaria in 2010 (with an uncertainty range of 154 million to 289 million) and an estimated 660 000 deaths (with an uncertainty range of 490 000 to 836 000) [1]. Malaria mortality rates have fallen by more than 25% globally since 2000 and by 33% in the World Health Organisation (WHO) African Region. Most deaths occur among children living in Africa where a child dies every minute from malaria [1]. Country-level burden estimates available for 2010 show that an estimated 80% of malaria deaths occur in just 14 countries and about 80% of cases occur in 17 countries [1]. Together, the Democratic Republic of the Congo and Nigeria account for over 40% of the estimated total of malaria deaths globally [1].

The artemisinins are new drugs developed from the Chinese wormwood (*Artemisia annua*), and the derivatives, namely, artemether, artesunate, and dihydroartemisinin, have now gained popularity as short-acting drugs which could be used in combination with drugs which have long life [2].

Malaria remains a major cause of morbidity and mortality in Nigeria in the era of improved control activities by the National Malaria Control Program (NMCP) since 2005. This is highly of concern and has necessitated the need to scale up interventions and assess the impact of malaria control measures in targeted areas. To implement effective interventions in an area, knowledge of malaria epidemiology and transmission dynamics, risk factors associated with malaria infection, and delay in diagnosis and treatment are of utmost importance. Many settings suitable for malaria vector propagation and prone to endemicity or resurgence of malaria have not been characterized in Lagos state, and Ibeshe is one of these settings.

Currently there is lack of data on parasite burden and inhabitants' knowledge, attitude and practices towards malaria control. Given its increasing population and coastal landmass that is amenable to malaria vector colonization and propagation, a malariometric survey of Ibeshe is highly needed to provide justification for expansion of malaria intervention settings in Lagos state.

## 2. Methodology

### 2.1. Ethical Approval

Ethical approval of the study was obtained from Nigerian Institute of Medical Research, Institutional Review Board (NIMR IRB).

### 2.2. Study Site

This cross-sectional study was carried out in the dry season, January 2011, in Ibeshe community (6° 30′ 26′′N, 3° 20′ 53E) in Ikorodu LGA, Lagos state. Convenient sampling method was adopted. Ibeshe is a semiurban community and is made up of 10 villages: Ibeshe central (Oluwoye, Ilemeri, and Orubo), Oshorun, Oke-Awori, Oke-Ota, Owode, Abuja, Agbowa, and Malatori. Ibeshe is about 20 km from Lagos metropolis. It has a population of about 23,850 people [3]. Their occupation consists primarily of peasant fishermen, farmers, and sand collectors. The community has 27 primary schools which consist of 2 public and 25 private schools. Only one public secondary school is in the community with one health centre. There are 4 private hospitals with two pharmacy stores. Numerous chemist stores are available in the community with seven traditional birth attendants.

Before the commencement of the study, the investigators visited the site and explain the study to the Oba of Ibeshe who gave his consent and permitted the town announcer to announce our mission to the community. He also obtained the cooperation of the chiefs (Baales) of the villages. The Baales of the villages visited provided us with the venue (canopy, chairs, and tables). Each site consisted of one or more villages. A total of 10 villages were covered during the exercise. Everybody in the community that came out for the survey were screened for fever (axillary temperature ≥37.5°C) and malaria parasite by microscopy, thick and thin malaria blood films stained with Giemsa stain. All persons that were positive for malaria parasite were treated with artemether-lumefantrine. Blood spots were made on filter paper for molecular studies on malaria parasite characteristics such as level of resistance to various antimalarial drugs.

The anaemia status of the people of Ibeshe was defined using the WHO haematocrit cutoff for mild anaemia Packed Cell Volume (PCV < 33%), moderate anaemia (PCV < 24%), and severe anaemia (PCV < 15%) [4]. Knowledge, attitude, and practices of the people towards malaria control were captured using interviewer-administered questionnaire. Statistical analysis was done with Epi-info 3.5.1.

## 3. Results

A total of 1489 participants were interviewed and screened for malaria and anaemia. The majority of the respondents were female 916 (62.3%). The mean age was 26.7 ± 20.0 years (range 0.1–99 years). Children under the age of 5 were 237 (16.6%), while those above 65 years of age were 73 (5.1%). The age group with highest population was 5–14 years 272 (19.0%). The respondents were mostly students 475 (32.6%) followed by traders 417 (28.6%). The famers were 16 (1.1%), and the lowest occupation was clergy 12 (0.8%). The number of respondents that attended secondary school was the highest 513 (40.7%), followed by primary 471 (37.4%). Only 36 (2.9%) respondents did not have any form of education. Majority of the respondents 731 (85.6%), who were 18 years and above, earn N20,000.00 or less per month. The major religions were Christianity 859 (59.9%) and Islam 551 (38.5%) (Table 1).

The prevalence of malaria in Ibeshe community was 14.7% (95% CI 13.0–16.6%). The prevalence of malaria in children aged 2–9 years was 16.4% (95% CI 12.6–21.2%). The predominant *Plasmodium* species found in the community was *Plasmodium falciparum* (93.6%). The geometric mean parasite density was 285 parasites per *μ*L of blood. The mean ± SD body temperature for participants with temperature ≥37.5 was 40 (2.7%). The participants that had normal PCV (≥33) were 1026 (81.9%), while those that had severe anaemia were 3 (0.2%) (Table 2).

The baseline characteristics of participants in different villages of Ibeshe community are shown in Table 3.

**Figure 1:**
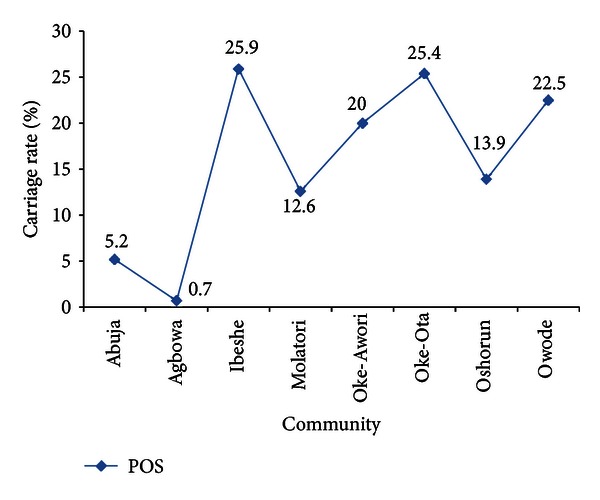
Malaria parasite carriage rate in Ibeshe community.

Figure 1 shows the malaria parasite carriage rate in the different villages; Agbowa and Abuja had the lowest malaria parasite rate, while Ibeshe and Oke-Ota had the highest rate.

**Figure 2:**
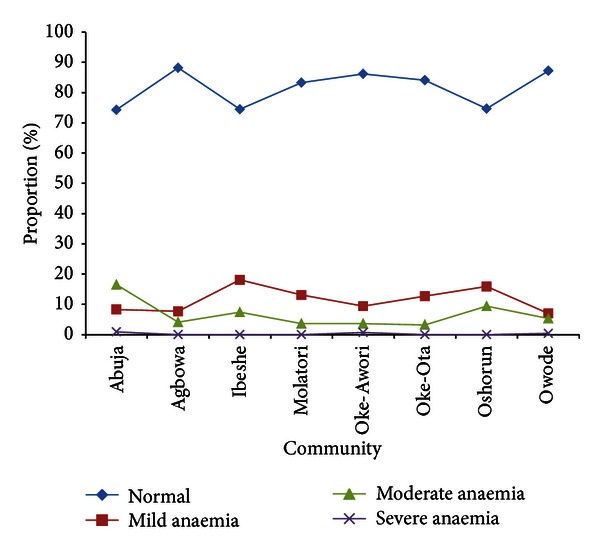
The anaemia status in the different villages of Ibeshe.

Figure 2 shows that majority of the participants in Ibeshe community had normal haemoglobin levels, mild anaemia was less than 20% in most of the villages.

There was no significant difference in comparing malaria positivity with temperature, sex, and age in the community (Table 4).

Parasite density above 500 parasites/*μ*L of blood was not associated with presence of fever in this study (Fisher's exact = 0.484).

The malaria infection rate was found to be higher in those aged 5 years and above (15.1%) than in those under 5 years of age (12.7%).

**Figure 3:**
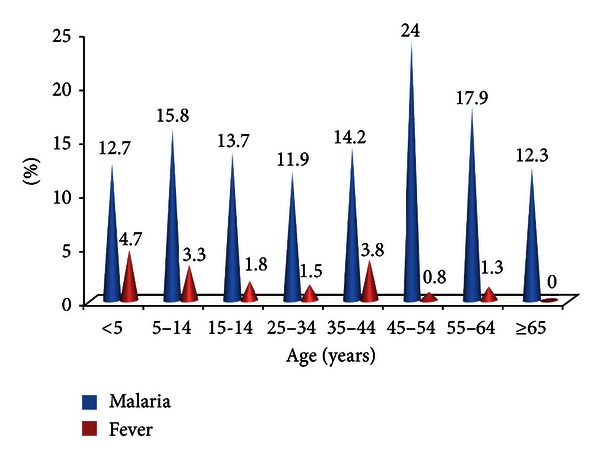
Proportion of febrile and malaria cases by age.

Figure 3 shows that febrile cases were highest in children <5 years (4.7%), while malaria infection rate was found to be highest in the age group between 45–54 years (24.0%).

**Figure 4:**
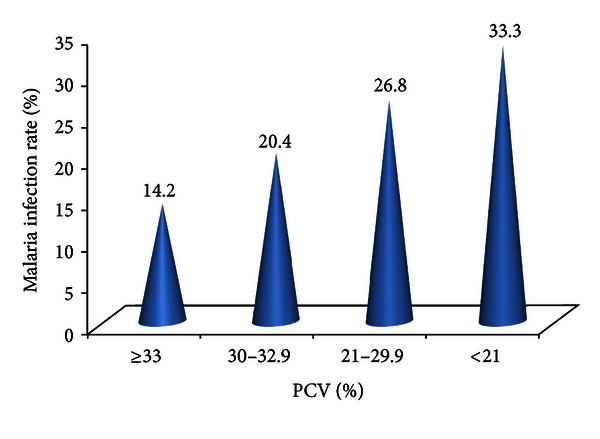
Relationship between malaria and level of PCV.

Figure 4 shows the significant relationship between malaria and level of PCV in the participants. The lower the PCV is, the higher the malaria infection rate observed.

Chi square = 12.37, *P* = 0.0006.

The respondents that reported fever in the past 24 hours were 1243 (83.5%). The number of respondent that consider the health facility too far from their home was 747 (50.2%), while 1021 (68.6%) were satisfied with treatment given at their health facilities. Some of the common suggestions on improving the health of people in Ibeshe were building more health facilities 1354 (90.9%), availability of drugs in health facilities 1302 (87.4%), erection of more public water taps 1300 (87.3%), and increasing the number of health workers 1274 (85.6%).

**Figure 5:**
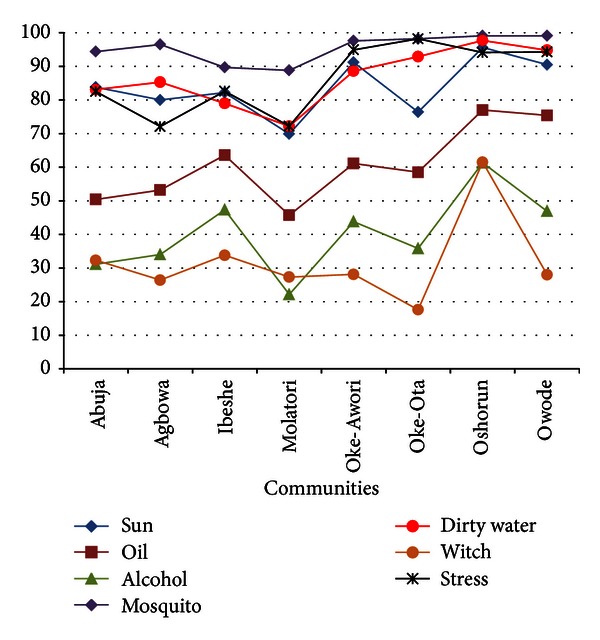
Knowledge of cause of malaria in the communities.

The respondents attributed the cause of malaria fever to be four major causes, in which mosquito bite (95.8%) is the major cause followed by dirty water (88.2%), while the other causes are working for too long (stress) (86.2) and staying in the sun (85.8); see Figure 5.

Majority of the respondents can recognize malaria symptoms by hot body (89.9%), headache (84.9%), refusal to eat (77.3%), and body ache (77.0). The respondents said that the action they will take when malaria occur would be to go to the hospital (65.6%), while 24.8% of the respondent would go to chemist/pharmacy. The respondents that would go to the traditional healers were (23.0%). The percentage of respondents that would treat at home was 22.5%; 15.0% of the respondents would either go to the church or mosques, while (0.5%) of the respondent would do nothing.

Most of the respondents lived less than 1 km from the health facilities (25.3%), while only 10.9% of the respondents lived above 10 km from the health facilities.

Measures taken for malaria protection, 77.0% of the respondents sleep with window nets, and 74.8% of the respondent clear bushes around them, while 65.1% clear their gutters. Only 29.6% of the respondents sleep under the long-lasting insecticidal net, while 27.7% sleep under the net.

Majority of the respondents spent greater than N1,500.00 on malaria treatment in a month, 385 (27.9%). Those that spent less than N500 per month were 290 (21.0%), while those that spent nothing per month were 208 (20.8%) (Table 5).

## 4. Discussion

Malaria control in Nigeria is essential; it is therefore necessary to know the burden of malaria in a community for planning and implementing appropriate interventions. The base line information on malaria and its control practices in an area enables the impact of malaria intervention programme to be measured. Good knowledge of behavior of people, as well as that of epidemiology of malaria, enhances correct prioritization of control strategies [5].

Ibeshe community can be classified as being mesoendemic for malaria at the time of this study based on the parasite rate in children aged 2–9 years old [6]. The malaria prevalence of 14.7% was reported in Ibeshe community in both children and adult in this study, of which the malaria prevalence in young adult (15–34 years) was 12.6%. A study carried out by Anumudu et al. [7] reported a prevalence of 17.0% in young adult (17–33 years) in a community in Ibadan, another area in southwestern Nigeria, which is higher than our result. This could suggest that malaria prevalence is reducing in southwestern Nigeria, probably due to the interventions employed by the Federal Government to controlling malaria in the region after the study carried by Anumudu et al. [7].

In this survey, malaria infection was observed to be associated with anaemia. This can be attributed to the ability of *P.  falciparum* to invade and destroy red blood cells at a more rapid rate than other human plasmodia parasites due to its greater virulence properties. The malaria infection rate was low in two villages (Agbowa and Abuja), and this could be due to a dead lake resulting from industrial pollution close to the villages.

The age group with the highest malaria infection rate was 45–54 years (24%), though most of them were not febrile. Children under the age of 5 years were the most febrile age group in this study, but their malaria infection rate was low, this is could be due to the fact that children under 5 years are known to have low immunity and are prone to other diseases that can cause fever. This study confirms the fact that it is not all fever cases that is caused by malaria infection.

The predominant species that was found in the community was* Plasmodium falciparum*, and this is consistent with Federal Ministry of Health report on *Plasmodium* species distribution in Nigeria [8]. There was no significant association between malaria parasite and body temperature as well as sex of the study participants. The parasite density in community was mostly low supporting the asymptomatic presentation observed in the community.

Knowledge about the cause of malaria is shrouded with a lot of misconceptions. Most of the respondents attributed the cause of malaria to more than one agent, the frequent responses being mosquito bite, sun, oil, and stress. Similar findings on the causes of malaria were reported in a study carried out in Akwa Ibom state by Ukpong et al. [9], where mosquito bite was reported to be the major cause of malaria followed by contaminated food and water. However, stress and sun were not reported as major causal factors of malaria in the Akwa Ibom study.

Our findings show that females outnumbered the males among the respondents; this is not surprising because this has been the normal pattern in most community studies carried out in many African disease endemic countries [10–12].

The success of malaria control programme at present relies on community perception of the disease; incorrect beliefs or inappropriate behavior can interfere with the effectiveness of a control measures such as vector control or chemotherapy [12].

The knowledge about malaria symptoms was high in the studied community. This is expected considering the high level of formal education and the endemicity of malaria in the area. High patronage of health facilities (hospitals and chemist/pharmacy) was report during malaria episodes in the community.

The use of long-lasting insecticide net in the community was low due to the fact that the rooms are hot because power supply is not regular, rather they prefer to screen their windows with net and environmental management. Oguonu et al. [13] also reported low usage of insecticide treated nets in rural and urban communities of Enugu, southeastern Nigeria.

Over 60% of the respondents aged 18 years and above earn less than N10,000.00 per month; this implies that the respondents were mostly in the low social economic class. It therefore means that malaria treatment is putting huge burden in the purse of the people in the community.

The implication of this study is that due to the intervention of malaria control programme, it has produced evidence of reduced malaria transmission and associated malaria burden in terms of parasite density in the studied area. Ibeshe community is one of the communities in Lagos state that had benefited from malaria control activities including long-lasting insecticide nets (LLINs) distribution, access to intermittent preventive treatment of malaria (IPT), and artemisinin-based combination therapies (ACTs) in the last 2 years.

However, the finding of this study suggests that malaria transmission is mesoendemic in this area. In a mesoendemic situation malaria is said to be unstable, identifying reservoirs of the parasite, and particularly under asymptomatic condition becomes critical in elimination of the parasite in the area. This study found participates of age group 45–54 years as reservoirs of *Plasmodium falciparum* in Ibeshe community. This is not unexpected since most of the interventions on malaria control are mainly on children under five years and pregnant women [8]. Based on the finding of this study, it is suggested that malaria control interventions should be extended to the adults in Ibeshe community.

### 4.1. Limitation of the Study

This study was carried out only in the dry session; it will be of interest to also carry out this study in the rainy session to ascertain the prevalence of malaria in the community.

## 5. Conclusion

Malaria is mesoendemic in Ibeshe community with *Plasmodium falciparum* being the predominant species. The participants had a good knowledge of the symptoms of malaria in the community; however, there are a lot of misconception on the cause of malaria. Anaemia is low in this community.

## 6. Recommendation

Enlightenment campaign is needed to change the people's perception on the cause of malaria for effective malaria control in the community. Focus on malaria control interventions should be extended to the adults in Ibeshe community.

## Figures and Tables

**Table 1:** Baseline characteristics of study participants.

Characteristic	*n* (%)
Sex	
Male	554 (37.7)
Female	916 (62.3)
Age (years)	
Mean ± SD (range)	26.7 ± 20.0 (0.1–99.0)
Age group	
<5	237 (16.6)
5–14	272 (19.0)
15–24	168 (11.7)
25–34	270 (18.0)
35–44	211 (14.8)
45–54	121 (8.5)
55–64	78 (5.5)
≥65	73 (5.1)
Weight (kg)	
Mean ± SD (range)	47.9 ± 26.3 (4.0–99.9)
Occupation	
Student	475 (32.6)
Trader	417 (28.6)
Artisan	182 (12.0)
Professional	73 (5.0)
House wife	56 (3.8)
Civil servant	35 (2.4)
Farmer	16 (1.1)
Clergy	12 (0.8)
Others	192 (13.2)
Education	
None	36 (2.9)
Primary	471 (37.4)
Secondary	513 (40.7)
Tertiary	102 (8.1)
Non formal	138 (11.0)
Income*	
None	238 (27.9)
<N10,000.00	342 (40.0)
N10,000.00–N20,000.00	151 (17.7)
N20,001.00–N30,000.00	48 (5.6)
N30,001.00–N40,000.00	29 (3.4)
N40,001.00–N50,000.00	15 (1.8)
>N50,000.00	31 (3.6)
Religion	
Christianity	859 (59.9)
Islam	551 (38.5)
Traditional	23 (1.6)

*This is for age 18 years and above.

**Table 2:** Malaria indicators.

Malaria	
Prevalence	219 (14.7)
*Plasmodium* species	
*P. falciparum *	205 (93.6)
*P. malariae *	12 (5.5)
Mixed (*P. falciparum + P. malariae*)	2 (0.9 )
Parasite density/*μ*L of blood	
Geomean	285
Range	21–221,714
1–500	161 (80.5)
501–1,000	24 (12.0)
1,001–5,000	11 (5.5)
>5,000	4 (2.0)
Axillary temperature (°C)	
Mean ± SD	36.6 ± 0.5
Range	35–40.3
≥37.5	40 (2.7)
<37.5	1431 (97.3)
PCV (%)	
Mean ± SD	36.3 ± 4.8
Range	18–50
PCV group	
Normal (≥33)	1026 (81.9)
Mild anaemia (30–32.9)	142 (11.3)
Moderate anemia (21–29.9)	82 (6.5)
Severe anaemia (<21)	3 (0.2)

**Table 3:** The baseline characteristic of participants in different villages in Ibeshe community.

	Abuja	Agbowa	Ibeshe	Malatori	Oke-Awori	Oke-Ota	Oshorun	Owode	*P*
*N*	192	269	239	103	150	63	237	236	
Mean age (±SD)	27.2 ± 18.8	22.5 ± 19.2	31.3 ± 23.2	30.8 ± 16.3	23.5 ± 17.7	25.2 ± 18.6	26.4 ± 20.8	27.3 ± 19.9	
<5 yrs (%)	27 (14.1)	56 (20.8)	31 (13.0)	7 (6.8)	27 (18.0)	13 (20.6)	37 (15.6)	39 (16.5)	0.07
Males (%)	75 (31.1)	117 (43.5)	77 (32.2)	44 (42.7)	55 (43.3)	28 (44.4)	75 (31.6)	73 (30.9)	0.002
Malaria carriage rate (%)	5.2	0.7	25.9	12.6	20.0	25.4	13.9	22.5	<0.001
Febrile cases (≥37.5°C)	6 (3.1)	9 (3.3)	10 (4.2)	1 (1)	1 (0.7)	2 (3.2)	4 (1.7)	7 (3.0)	0.411

**Table 4:** Malaria positivity comparing temperature, sex, and age.

Character	Malaria positivity *n* (%)	*P*
Temperature (°C)		
<37.5	210 (14.7)	0.17
≥37.5	9 (22.5)	
Sex		
Male	85 (15.3)	0.625
Female	132 (14.4)	
Age (years)		
<5	30 (12.7)	0.319
≥5	181 (15.2)	

**Table 5:** Knowledge and practice of malaria control.

	*n* (%)
Cause of malaria fever*	
Staying in the sun	1089 (85.8)
Oil	474 (63.6)
Alcohol	500 (43.5)
Mosquito bite	1292 (95.8)
Dirty water	1087 (88.2)
Witchcraft	391 (34.5)
Working for too long (stress)	1065 (86.2)
Recognition of malaria symptoms*	
Hot body	133 (89.9)
Vomiting	877 (58.9)
Refusal to eat	1151 (77.3)
Body ache	1146 (77.0)
Headache	1264 (84.9)
Diarrhea	628 (42.2)
Sweating	1030 (69.2)
Fatigue	998 (67.0)
Malaise	978 (65.7)
Sleeping all day	478 (32.1)
Dull	421 (28.3)
Bitter taste	391 (26.3)
Yellow urine	365 (24.5)
Action taken when malaria occur (*n* = 1243)*	
Treat at home	280 (22.5)
Go to chemist/pharmacy	308 (24.8)
Go to hospital	815 (65.6)
Go to church/mosque	15 (1.2)
Go to traditional healer	23 (1.2)
Do nothing	6 (0.5)
Distance to health facility (*n* = 1225)	
<1 km	310 (25.3)
1-2 km	238 (19.4)
>2–5 km	283 (23.1)
>5–10 km	260 (21.2)
>10 km	134 (10.9)
Malaria protective measures taken*	
Sleeping under the net	413 (27.7)
Sleeping under insecticide treated net	441 (29.6)
Sleeping with windows closed	752 (50.5)
Sleeping with window with net	1147 (77.0)
Use of insecticide spray (Shelltox, Raid, Baygon)	854 (57.4)
Burning coil/grass	765 (51.4)
Clearing bushes	1114 (74.8)
Draining stagnant water	897 (60.2)
Clearing gutter	969 (65.1)
Covering the body with cloth	784 (52.7)
Amount spent on malaria treatment in a month (*n* = 1379)	
None	287 (20.8)
<N500.00	290 (21.0)
N500.00–N1,000.00	271 (19.7)
>N1,000.00–N1,500.00	146 (10.6)
>N1,5000.000	385 (27.9)

*Multiple responses.
